# Primary hepaticobiliary tuberculosis mimicking gall bladder carcinoma with liver invasion: a case report

**DOI:** 10.11604/pamj.2019.32.68.10519

**Published:** 2019-02-07

**Authors:** Muhammad Manzoor Ul Haque, Rajesh Kumar Whadva, Nasir Hassan Luck, Muhammed Mubarak

**Affiliations:** 1 Department of Hepatogastroentrology, Sindh Institute of Urology and Transplantation (SIUT), Karachi, Pakistan; 2 Department of Histopathology, Sindh Institute of Urology and Transplantation (SIUT), Karachi, Pakistan

**Keywords:** Carcinoma, frozen section, gall bladder, liver biopsy, tuberculosis

## Abstract

Primary hepatic tuberculosis is a rare presentation and sporadically reported in the literature, mostly from our part of the world. Sometimes the presentation can be atypical and mimics hepatic tumor and poses diagnostic challenge. We, herein, present a case of a 58-year-old man who presented to us with abdominal pain and weight loss. Raised serum alkaline phosphatase (ALP) and imaging raised a suspicion of gall bladder carcinoma with hepatic invasion. Peroperative frozen section revealed hepatic chronic granulomatous inflammation with caseous necrosis consistent with the diagnosis of hepatic tuberculosis. Surgery was postponed and he was put on antituberculous treatment. It is important to consider tuberculosis in the differential diagnosis of the space occupying lesion of liver in a patient with vague symptoms and abnormal findings on imaging.

## Introduction

Tuberculosis (TB) is a worldwide health problem with particularly high prevalence in developing countries [1, 2]. The worldwide burden of TB is estimated to be 8.6 million incident cases in year 2012; the South East Asia and western pacific region are among the most prevalent areas accounting for 58% of world TB cases [3]. Abdominal TB is one of the most prevalent forms of extrapulmonary disease [4]. Hepatic involvement occurs in less than 1% of all cases and usually it is associated with foci of infection in the lungs or gastrointestinal tract [5, 6]. Primary hepaticobiliary TB is considered very rare among abdominal TB. It is sporadically reported in the literature mostly from our part of the world. It can have atypical presentation and mimic hepatic tumor, posing diagnostic challenge to the clinicians. Herein, we report a case of a middle aged male who presented to our hepatogastroenterology clinic with abdominal pain, vomiting and weight loss and was found to have raised alkaline phosphatase (ALP). The abdominal imaging studies raised a suspicion of gall bladder (GB) carcinoma with hepatic invasion. However, peroperative frozen section revealed hepatic epithelioid cell granulomata with caseous necrosis consistent with TB.

## Patient and observation

**Figure 1:**
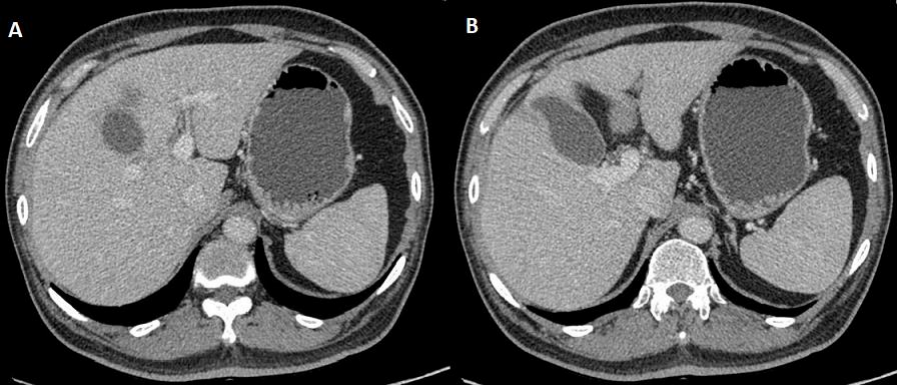
Computerized tomographic (CT) images of the case: A) gall bladder showing diffusely thickened wall and irregularities at fundus; B) surrounding liver shows small area of hypodensity suggestive of gall bladder malignancy with liver metastasis

**Figure 2:**
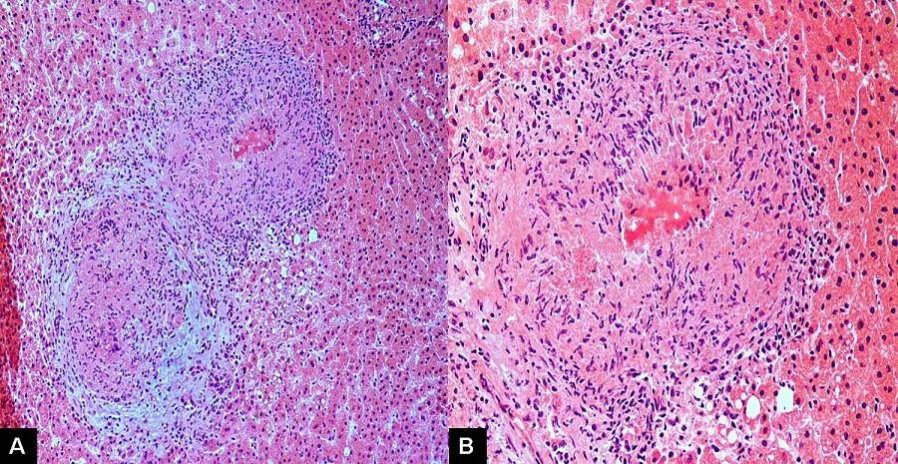
Histopathological images: A) low-power view showing two well-formed epithelioid granulomata with central necrosis (hematoxylin and eosin, ×100); B) medium-power view showing well-formed epithelioid granuloma with central caseation necrosis (hematoxylin and eosin, x 200)

A 58-year-old man presented to our hepatogastroenterology clinic with non-colicky pain in right hypochondrium and vomiting for 1 month. The pain was mild in intensity, dull, aching in nature, gradual in onset and progressive in course. It was non-radiating and not associated with meals. There was history of weight loss of 3 kg over a period of 1 month. There was no history of jaundice and tuberculosis or exposure to tuberculosis in the past. The general physical examination was unremarkable. There was no lymphadenopathy or abdominal visceromegaly. The laboratory investigations revealed raised ALP, 111 IU/L, Gamma-glutamyl transpeptidase (GGT), 61 IU/L and erythrocyte sedimentation rate (ESR), 70 mm/1^st^h; while complete blood count (CBC), renal function tests, coagulation profile and CA 19-9, 18.9u/ml (normal: 0-31u/ml) were within normal limits. Ultrasonography (US) of the abdomen revealed distended and irregular thick walled GB. Computed tomography (CT) of the abdomen revealed GB with irregularly thickened wall in the region of the fundus. There was an illness defined hypodense area extending from the body of GB to adjacent hepatic parenchyma measuring 3.0x1.5 cm and an enlarged lymph node at porta hepatis measuring 2.0x1.5 cm. The above constellation of findings raised a radiological suspicion of malignant lesion of GB (Figure 1). Bone scintigraphy and CT chest were negative for metastatic deposits. Based on the clinicoradiological diagnosis of GB carcinoma, extended cholecystectomy was planned. Diagnostic laparoscopy was done to get tissue diagnosis prior to the definitive procedure. It revealed adhesions around liver with multiple small whitish spots on its surface. Liver lesion biopsy was taken as well as two lymph nodes removed and sent for frozen section. The histopathological evaluation of frozen section of liver tissue revealed numerous caseating epithelioid granuloma containing Langhan’s giant cells, consistent with tuberculosis (Figure 2). The sections examined from lymph nodes revealed benign reactive changes. Based on these findings, exploratory laparotomy was not done and the wound was closed. Patient was prescribed anti-tuberculous treatment (ATT) for one year. After 4 months of starting ATT, the patient improved markedly. His clinical symptoms completely disappeared. Repeated laboratory investigations revealed ESR 10 mm/1^st^h, and liver function tests (LFTs) also returned to normal levels. He is still on treatment and on regular follow-up.

## Discussion

Hepatic TB as a part of disseminated TB is seen in 50-80% of cases. However, isolated or primary localized hepatic TB is very uncommon even in countries with high prevalence of the disease. The isolated liver TB without active pulmonary or miliary TB, or other clinical evidence of TB can easily be misdiagnosed in clinical practice, and only few cases have been reported in the literature [7]. Hepatobilliary TB occurs most commonly as a part of miliary tuberculosis due to hematogenous dissemination; however, it may also spread through the lymphatic and portal system. One reason for the rarity of the entity is the unfavorable environment for the growth of mycobacteria in low oxygen tension [8-10].

Classically, there are three patterns of hepatic involvement in TB i.e. miliary hepatic, nodular hepatic and biliary tract TB [2]. The first two involve the hepatic parenchyma while the later one involves the biliary tree. The clinical presentation of hepatic TB is most often subtle and insidious. Usually, it presents with nonspecific symptoms including fever and hepatomegaly along with constitutional symptoms such as abdominal pain, anorexia and weight loss. Elevated serum levels of ALP and liver enzymes may be observed particularly in biliary TB [8, 11, 12]. Imaging studies may detect a solitary space occupying lesion in the liver, manifesting as hypoechoic mass on sonography and a hypodense mass with or without hyperdense rim on CT scan [9-12]. As such, hepatic tuberculoma can mimic hepatocellular carcinoma or hepatoma.

In our case, the patient presented with abdominal pain and weight loss. His ALP was raised. The imaging studies showed distended and irregularly thick walled GB on ultrasound, while contrast enhanced CT revealed thickening of the GB wall at the fundus region with a soft tissue density and reported as GB carcinoma by the radiologist. The definitive diagnosis of hepatic TB requires pathological and bacteriological evidence. Due to the low yield of culture and acid fast bacilli (AFB) staining, CT/US guided percutaneous biopsy or peroperative biopsy is the preferred approach for the diagnosis. Diaz *et al.*, reported the sensitivity of mycobacterial antigen PCR up to 57% for hepatic granuloma [5]. In our case, AFB staining of the paraffin embedded hepatic tissue sections was negative but AFB PCR was found positive.

Regarding management of the condition, most often surgical intervention is done due to pre-operative diagnosis of malignancy rather than tuberculosis. As in our case, on the basis of clinicoradiological diagnosis of GB carcinoma, the patient was planned for extended cholecystectomy. Peroperatively, we found multiple whitish spots on the surface of liver, whose frozen section revealed epithelioid granulomata with central necrosis. Two lymph nodes were also sent for histopathology, which revealed reactive changes. The frozen section histopathology spared the patient an unnecessary radical surgery.

Once the diagnosis of hepatic TB is established, conventional ATT for 1 year will cure the disease nearly in 100% of cases. In biliary TB causing obstruction of the biliary tree, there may be a need for ERCP (Endoscopic Retrograde Cholangio-Pancreatography) to relieve the obstruction. Tuberculosis should be kept in the differential diagnosis of space occupying lesions of the liver, especially in endemic areas. Our patient has been given ATT and is on follow up; he was found to be afebrile and had recent weight gain of 4 kg in a period of 4 months of therapy.

## Conclusion

Isolated hepaticobililary TB is a rare entity with potentially curable outcome, but presents a diagnostic challenge due to its atypical presentation. It is necessary, to consider it in the differential diagnosis of space occupying lesions of the liver in endemic areas.

## Competing interests

The authors declare no competing interests.
